# Determinants of first-line biological treatment in patients with rheumatoid arthritis: Results from an observational study

**DOI:** 10.1097/MD.0000000000025943

**Published:** 2021-05-14

**Authors:** Laura Angelici, Antonio Addis, Nera Agabiti, Ursula Kirchmayer, Marina Davoli, Valeria Belleudi

**Affiliations:** Department of Epidemiology, Lazio Regional Health Service, Rome, Italy.

**Keywords:** arthritis rheumatoid, biologic, first-line therapy, switching

## Abstract

Guidelines for the treatment of rheumatoid arthritis (RA) recommend the use of conventional synthetic disease modifying anti-rheumatic drugs (cs-DMARDs) at the onset of the disease and only in the case of therapeutic failure, the addition of a biological drug (b-DMARD) is suggested.

The study aimed to evaluate determinants for first-line biological treatment in patients with RA in clinical practice.

A cohort of patients with RA, resident in Lazio, a central Italian Region, where Rome is located, and with at least one disease modifying anti-rheumatic drugs (DMARD) prescription between 2010 and 2016 was selected using health information systems linkable with each other by an individual unique anonymous identifier. In particular RA cohort was defined retrieving all patients with at least a RA disease code in regional data claims (hospital discharge, exemption code, emergency department access, or therapeutic plan). Only new users were included and the first-line treatment was identified: cs-DMARD or b-DMARD.

Descriptive analysis according to type of DMARD treatment was performed. Through multivariate logistic regression models (odds ratio [OR]; confidence interval [CI95%]) determinants of therapy such as age, comorbidity, and comedication were investigated.

Finally, switching during the first year of treatment from cs-DAMARDs to b-DMARDs was analyzed.

DMARD-new users with RA were 5641; 7.1% of them with b-DMARD as first-line treatment. Considering the year of dispensing, this percentage ranged from 4.9% (2011) to 8.2% (2015). Among cs-DMARD the most prescribed active agent was methotrexate (59.3%), while among b-DMARD it was etarnecept (37.0%), followed by adalimumab (21.2%). The average age of the cohort was 54 years with 77% of women. Determinants of first-line b-DMARD use were: age (OR_<30vs>65_ = 3.7; 2.6–5.2, OR_[30–45)vs>65_ = 1.7; 1.2–2.4, OR_[45–55)vs>65_ = 1.6; 1.1–2.4, OR_[55–65)vs>65_ = 1.2; 0.8–1.7), cancers (OR = 2.3; 1.3–4.2), cardio-cerebrovascular disease (OR = 1.4; 1.0–1.9), use of non-steroidal anti-inflammatory drugs (NSAIDs) (OR = 0.6; 0.4–0.7) and corticosteroids (OR = 0.6; 0.5–0.7) in the 6 months preceding diagnosis.

In the first year of treatment, we observed a percentage of switch from cs-DMARDs to b-DMARDs of 7.9%.

In clinical practice, about 7% of patients with RA are prescribed with a b-DMARD as first-line treatment. This therapeutic option, even if not supported by guide lines, is mostly link to younger age and clinical profile of the patients.

**Key Points**

- Guidelines for the management of RA recommend the use of biologics (b-) after failing conventional DMARDs (cs-).
- In clinical practice, about 7% of patients with RA are prescribed with a b-DMARD as first-line treatment, this therapeutic option is mostly associated with younger age.
- The decision to administer biologics in DMARD-naïve patients among younger patients could reflect a perceived difference in efficacy between cs- and b-DMARDs.
- Further analyses on real world data are necessary to investigate appropriateness and to promote a better use of biologics in RA.

## 1 Introduction

Rheumatoid arthritis (RA) is a chronic autoimmune condition affecting synovial joints, with a lifetime prevalence of up to 1% worldwide.^[1]^ The prevalence is estimated to be 2/3 times higher in women than men.^[2]^ Onset can occur at any age, but peaks between 30 and 50 years.^[3]^ The consequence is irreversible damage to joint tissues a few months after onset, resulting in joint deformity within few years.

Without treatment, the inflammatory process leads to joint destruction, pain, deformity, disability, and cardiovascular diseases. The discovery and introduction of new therapies, for example, biologic and targeted synthetic, have dramatically improved the management of RA, revealing as a valid therapeutic alternative for patients who have inadequate response to conventional synthetic disease-modifying antirheumatic drugs (cs-DMARDs).^[4]^

In fact, the current standard treatment of RA recommends the use of cs-DAMRDs, mostly methotrexate, as a first-line strategy.^[5,6]^ In cases of non-response, add on therapies with new DMARDs (e.g., biologics or Janus kinase inhibitors, also known as JAK inhibitors) are considered effective.^[7–9]^ In particular, available evidence shows that combination therapy (biologic plus conventional synthetic DMARDs) is in general more efficacious than biological monotherapy.^[10]^

Although several randomized clinical trials (RCT) have demonstrated the efficacy of these therapies, data on direct comparison between biologics and conventional drugs, and among different biologics in terms of safety and effectiveness (short and long term) are scarce.^[11–13]^

In clinical practice, following overarching principles reported in the 2019 updated European Alliance of Associations for Rheumatology (EULAR) RA management recommendations,^[9]^ the choice of treatment for a patient should be influenced by patient-specific factors such as duration and severity of the disease, previous treatments, comorbidities as well as by other aspects such as costs and way of administration. In this context, the decision about which drug to choose as first-line therapy could reflect, to a certain extent, perceived differences in safety and efficacy across drugs.

Monitoring prescribing patterns in clinical practice, using real world data, is a necessary step to increase understanding of the impact of therapeutic choice on patients and to evaluate the adherence to guidelines. In Italy, a growing number of healthcare databases, collecting information on outpatients (including dispensing of drugs, diagnostic tests/specialist examinations, etc) and inpatients (including inpatient medical procedures, drug dispensing, etc) care covered by the National Health System, have been used to evaluate post-marketing drug utilization and safety in the last 2 decades.^[14]^ The aims of this study were to describe the use of biologic DMARDs (b-DMARDs) as first-line treatment, to analyze its determinants in a real-world cohort of DMARD-naive patients with RA and to measure the switch from cs-DMARDs to b-DMARDs in the first year of treatment.

## 2 Methods

### 2.1 Study design and data sources

We conducted an observational, record-linkage, multi-database, retrospective cohort study using fully anonymized data from Lazio region (about 6 million of inhabitants) health administrative databases. In particular, to select the study population and track subjects’ eligibility during follow-up individual-level information was retrieved from: regional population register receiving National Health Service assistance (PR), regional drug claims register (RDCR), hospital discharges register (HIS), emergency department (ED) visits register, disease-specific payment exemptions register (DPER), data from outpatient clinics, and electronic therapeutic plan register for biological prescriptions. The RDCR, collecting information on drug prescriptions reimbursed by the healthcare system and dispensed by private or public pharmacies (including hospital pharmacies), were also used to determine drug consumption, while HIS along with DPER were used to determine comorbidities. Finally, death certificates from the regional mortality register were used to update the PR. All data sources can be linked using anonymous keys. All residents of the Lazio region enrolled in the public health service have a personal identification number recorded in all regional healthcare databases. This individual identifier provides the key to link all regional databases and allows to identify individuals uniquely within the regional health system. The anatomical therapeutic chemical (ATC) classification system was used to code drugs, while the International Classification of Disease, Clinical Modification, Ninth Revision (ICD9-CM) was used to code diseases in relation to healthcare services payment exemption, diagnoses at hospital discharges, and reasons for ED visits.

All described healthcare claims are frequently used in pharmacoepidemiology research to generate post-marketing evidence on drug use and safety.^[14]^

The ethical approval was not required because the study uses administrative date and was conducted with the permission of the Department of Epidemiology of Lazio Regional Health Service, the regional referral center for epidemiological research who has full access to anonymized data. The Department of Epidemiology is legitimised by the Lazio Region in managing and analysing data for epidemiological purposes. This study was carried out in full compliance with the current privacy laws.

### 2.2 Study population

RA cohort was defined selecting all resident in the Lazio region between January 1, 2010, and December 31, 2016 with at least one hospital admission or emergency department visit or electronic therapeutic plan (ICD-9-CM codes 714.0, 714.1, 714.2, 714.30, 714.30, 714.32, 714.33) or registered with a disease exemption for RA (code 006). The overall validity of the Italian administrative databases in the identification of patients with RA has been previously demonstrated.^[15]^ From this population we selected only patients with at least one prescription for DMARD from January 1, 2010, to December 31, 2016 (index date) and with a diagnosis of RA prior to the prescription of DMARD. Moreover, exclusion criteria were as follows: not being registered in the regional health care system during the study period, diagnosis of connective tissue disease, or discharge with a diagnosis of connective tissue disease or inflammatory bowel disease (IBD) with indications for DMARDs therapy within the 36 months prior to the index date.

### 2.3 Outcome

Patterns of use of DMARDs available on the market in the study period were evaluated in terms of first-line cs-DMARDs therapy or first-line b-DMARDs therapy (included combined therapy).

We defined the index date as the date of the first prescription of any study drug. Only new users of the drug were included, considering a 12-month washout period before the index date during which the patient did not use any DMARD. The time window chosen to define new users is commonly used in observational research.^[16]^ Each patient was followed from index date until death or 1 year, whichever came first.

### 2.4 Demographic and clinical characteristics

Demographic characteristics were determined from PR, coexisting conditions were determined from hospital discharges register and DPER, drug consumption was determined from RDCR. Patient's age at index date was classified in 5 classes of years: <30; 30 to 44; 45 to 54; 55 to 64; ≥65. The following comorbidities were retrieved from HIS within 36 months before the index date: cancer, conduction disorder and arrhythmias, cardio-cerebrovascular disease, psychiatric disease, chronic obstructive pulmonary disease and respiratory failure, neuromuscular disease, liver, pancreas and kidney disease, arthrosis.

The following chronic comorbidities were retrieved from HIS, DPER, and RDCR within 36 months before the index date: diabetes, hypertension, hypothyroidism. Infections and pneumonia were retrieved from HIS 6 months before the index date.

Concomitant drug utilization, in terms of number of drug prescriptions in the 6 months prior to the index date, for the following pharmacological treatments, indicated for RA: non-steroidal anti-inflammatory drugs (NSAIDs), corticosteroids, analgesics, opioids, psychoanaleptics, antiepileptics was considered.

### 2.5 Statistical analysis

Descriptive analysis according to type of DMARD treatment (cs-DMARDs first-line, b-DMARDs first-line, or combination) was performed for demographic characteristics (sex and age), concomitant conditions and concomitant drug utilization. We investigated the use of cs-DMARDs first-line treatment, b-DMARDs first-line, or combination in the selected cohort according to type of drug and by year of prescription.

Differences in demographic and clinical characteristics between cs-DMARDs and b-DMARDs initiators were described, in terms of number and percentage of new users, and tested by chi-square test. Logistic regression models (OR; CI95%) were used to investigate the role of demographic characteristics, clinical history, and comedications as potential determinants of first-line b-DMARDs respect to first-line cs-DMARDs or combination. Among all factors potentially associated with the outcomes under study, age, sex, and arthrosis were considered as a priori risk factors; the others were selected by stepwise logistic regression. The analysis was carried out for the whole population and stratifying by sex.

Sensitivity analysis was performed removing patients with cancer from the model in order to evaluate a possible misclassification of indication for rituximab.

Finally, switching during the first year from cs-DAMARDs to b-DMARDs was investigated in terms of time and number of switches. A chord diagram was built to visualize patterns of switch in the first year after the RA onset.

## 3 Results

### 3.1 Patients characteristics

**Figure 1:**
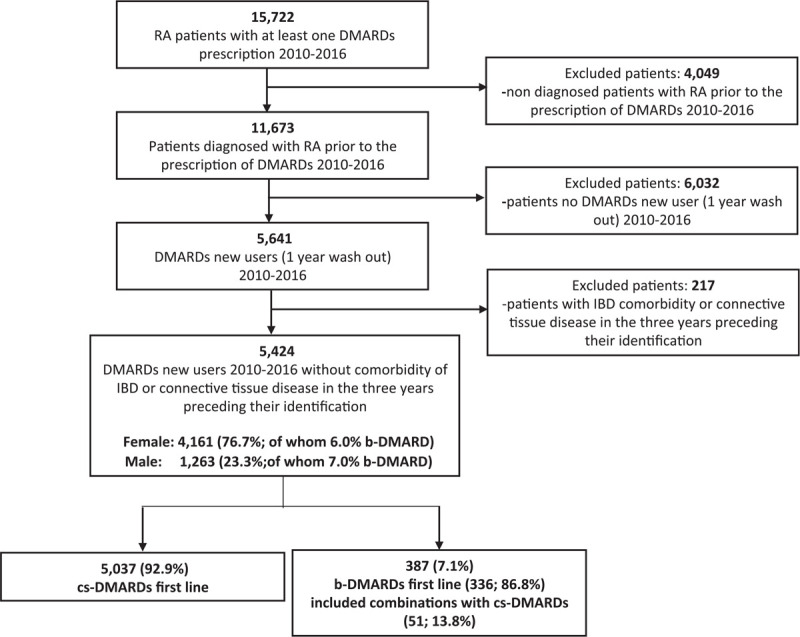
Flow chart for cohort selection.

In the Lazio Region, we observed a cohort of 15,722 patients with RA between 2010 and 2016 (Fig. 1); in this population, 11,673 had at least one prescription for DMARDs after the diagnoses of RA. The percentage of DMARD new users was 48.3% (N = 5641). After exclusion of patients with comorbidity of IBD or connective tissue disease in the 3 years preceding their identification by HIS, DMARD new users with RA were 5424; 92.9% (N = 5037) of them were treated with cs-DMARDs as first-line treatment, 7.1% (N = 387) with b-DMARDs as first-line treatment (13.8% of whom in combination with cs-DMARDs).

Among cs-DMARDs the most prescribed active agent was methotrexate (59.2%), while among b-DMARD it was etarnecept (32.4%), followed by adalimumab (22%) (Table 1).

**Table 1:** Distribution of cs-DMARD first-line, b-DMARD first-line, and combination by active agent in DMARD new users.

	cs-DMARDfirst-line			b-DMARDfirst-line				Total	
				Alone		In combination with cs			
Active agents	ATC	N	%	N	%	N	%	N	%
Methotrexate	L01BA01/L04AX03	2985	59.3					2985	55.0
Hydroxycloroquine	P01BA02	1337	26.5					1337	24.7
Sulfasalazine	A07EC01	533	10.6					533	9.8
Leflunomide	L04AA13	182	3.6					182	3.4
Etanercept	L04AB01			109	32.4	34	66.7	143	2.6
Adalimumab	L04AB04			73	21.7	9	17.7	82	1.5
Rituximab	L01XC02			43	12.8	0	0.0	43	0.8
Anakinra	L04AC03			31	9.2	3	5.9	34	0.6
Tocilizumab	L04AC07			25	7.4	4	7.8	29	0.5
Abatacept	L04AA24			17	5.1	0	0.0	17	0.3
Certolizumabpegol	L04AB05			15	4.5	1	2.0	16	0.3
Infliximab	L04AB02			9	2.7	0	0.0	9	0.2
Golimumab	L04AB06			14	4.2	0	0.0	14	0.3
Total		5037	100.0	336	100.0	51	100.0	5424	100.0

b-DMARDs = biological-DMARDs, cs-DMARDs = conventional synthetic-DMARDs, DMARDs = disease modifying anti-rheumatic drugs.

### 3.2 Main results

**Figure 2:**
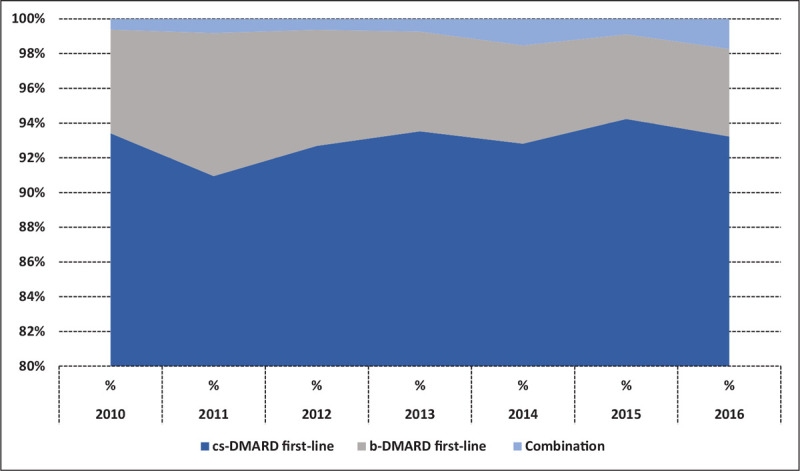
Distribution of cs-DMARD first-line, b-DMARD first-line and combination by year of dispensing in DMARDs new users. b-DMARDs = biological-DMARDs, cs-DMARDs = conventional synthetic-DMARDs, DMARDs = disease modifying anti-rheumatic drugs.

Considering the year of dispensing, the percentage of b-DMARD first line users ranged from 4.9% (2011) to 8.2% (2015) (Fig. 2). However, in the study period the percentage of prescriptions of etarnecept for RA patients decreased from 3.7 in 2010 to 1.7 in 2016.

The average age of the cohort was 54 years with 77% women; the prevalence of diseases such as hypertension, diabetes, hypothyroidism, cerebrocardiovascular diseases, arthrosis, and osteoporosis was 43.5%, 18.8%, 16.7%, 12.0%,6.7%, 7.9% respectively. In the 6 months preceding the start of therapy, 61.4% used NSAIDs and 62.1% corticosteroids (Table 2).

**Table 2:** DMARD new users’ characteristics according to first-line therapy with cs-DMARDsor b-DMARDs (included combinations).

	cs-DMARD first-line5037		b-DMARD first-line387		Total5424	
	N	%	N	%	N	%
Sex						
Female	3873	76.9	288	74.4	4161	76.7
Male	1164	23.1	99	25.6	1263	23.3
Age categories^∗^						
<30 years	600	11.9	124	32.0	724	13.3
30–44 years	863	17.1	67	17.3	930	17.1
45–54 years	991	19.7	70	18.1	1061	19.6
55–64 years	1259	25.0	65	16.8	1324	24.4
≥65 years	1324	26.3	61	15.76	1385	25.5
Cancer^∗^	103	2.0	13	3.36	116	2.1
Conduction disorders and arrhythmias	116	2.3	5	1.3	121	2.2
Cardio-cerebrovascular diseases^∗^	608	12.1	44	11.4	652	12.0
Psychiatric diseases (dementia, Alzheimer, depression)	43	0.9	2	0.5	45	0.8
COPD and respiratory failure	82	1.6	4	1.0	86	1.6
Neuromuscular diseases	27	0.5	3	0.8	30	0.6
Liver, pancreas, and kidney diseases	64	1.3	6	1.6	70	1.3
Arthrosis^∗^	329	6.5	34	8.8	363	6.7
Infections	24	0.5	2	0.5	26	0.5
Diabetes^∗^	964	19.1	53	13.7	1017	18.8
Hypertension^∗^	2231	44.3	127	32.8	2358	43.5
Hypothyroidism	849	16.9	55	14.2	904	16.7
Osteoporosis^∗^	410	8.1	21	5.4	431	7.9
NSAIDs^∗^	3171	63.0	161	41.6	3332	61.4
Corticosteroids^∗^	3203	63.6	166	42.9	3369	62.1
Analgesics	645	12.8	39	10.1	684	12.6
Opioids	608	12.1	38	9.8	646	11.9
Psychoanaleptics	400	7.9	24	6.2	424	7.8
Antiepileptics	291	5.8	19	4.9	310	5.7

COPD = chronic obstructive pulmonary disease.

∗ Results of Chi square test show differences statistically significant (*P* < .05).

Determinants of first-line b-DMARD use were: age (OR_<30vs>65_ = 3.7; 2.6–5.2, OR_[30–45)vs>65_ = 1.7; 1.2–2.4, OR_[45–55)vs>65_ = 1.6; 1.1–2.4, OR_[55–65)vs>65_ = 1.2; 0.8–1.7), cancers (OR = 2.3; 1.3–4.2), cerebrocardiovascular disease (OR = 1.4; 1.0–1.9), arthrosis (OR = 0.7; 0.5–1.1), use of NSAID (OR = 0.6; 0.4–0.7), and corticosteroids (OR = 0.6; 0.5–0.7) in the 6 months preceding the diagnosis (Table 3).

**Table 3:** Multivariable logistic regression with independent determinants associated to first-line therapy with b-DMARD versus cs-DMARD.

Main analysis				
Determinant for b-DMARD first-line	OR	95% CI		*P*-value
Sex _F__vs M_	0.86	0.68	1.10	.2377
Age _<30 vs >65_	3.66	2.56	5.23	<.0001
Age [_30–45) vs >65_	1.68	1.15	2.44	.0068
Age [_45–55) vs >65_	1.64	1.14	2.37	.0079
Age [_55–65) vs >65_	1.18	0.82	1.69	.3824
Cancer	2.30	1.25	4.24	.0074
Arthrosis	0.71	0.48	1.07	.1012
Cardio-cerebrovascular diseases	1.36	0.96	1.94	.0839
Non-steroidal anti-inflammatory drugs	0.58	0.46	0.73	<.0001
Corticosteroids	0.56	0.45	0.70	<.0001

b-DMARDs = biological-DMARDs, CI = confidence intervals, cs-DMARDs = conventional synthetic-DMARDs, OR = odds ratio.

### 3.3 Subgroup and sensitivity analyses

The sensitivity analysis after removing cancer from the selected determinants in the final model did not show relevant changes, except for cardio-cerebrovascular diseases that became a statistically significant factor (Table 4).

**Table 4:** Multivariable logistic regression with independent determinants associated to first-line therapy with sensitivity analysis for multivariable logistic regression removing cancer.

Sensitivity analysis				
Determinant for b-DMARD first-line	OR	95% CI		*P*-value
Sex _F vs M_	0.86	0.67	1.10	.2184
Age _<30 vs >65_	3.51	2.46	5.00	<.0001
Age [_30–45) vs >65_	1.61	1.11	2.34	.0117
Age [_45–55) vs >65_	1.58	1.10	2.28	.0132
Age [_55–65) vs >65_	1.15	0.80	1.65	.4598
-	-	-	-	-
Arthrosis	0.71	0.48	1.07	.0996
Cardio-cerebrovascular diseases	1.43	1.01	2.02	.0455
Non-steroidal anti-inflammatory drugs	0.58	0.46	0.73	<.0001
Corticosteroids	0.56	0.45	0.70	<.0001

b-DMARDs = biological-DMARDs, CI = confidence intervals, OR = odds ratio.

Stratified analysis by sex showed that the association pattern was similar between men and women: age (M: OR_<30vs>65_ = 5.9; 3.0–11.9, OR_[30–45)vs>65_ = 2.2; 1.0–4.9, OR_[45–55)vs>65_ = 2.3; 1.1–4.8, OR_[55–65)vs>65_ = 0.5; 0.2–1.2; F: OR_<30vs>65_ = 3.0; 2.0–4.5, OR_[30–45)vs>65_ = 1.5; 1.1–2.3, OR_[45–55)vs>65_ = 1.5; 1.2–2.3, OR_[55–65)vs>65_ = 1.4; 0.9–2.1), cancers (M: OR = 1.6; 0.4–5.7; F: OR = 2.5; 1.3–5.1), cardio-cerebrovascular disease (M: OR = 1.4; 0.7–2.9; F: OR = 1.4; 0.9–2.1), arthrosis (M: OR = 0.; 0.1–0.7; F: OR = 0.9; 0.6–1.5), use of NSAID (M: OR = 0.4; 0.3–0.7; F: OR = 0.6; 0.5–0.8) and corticosteroids (M: OR = 0.4; 0.3–0.7; F: OR = 0.6; 0.5–0.8).

### 3.4 Switching

**Figure 3:**
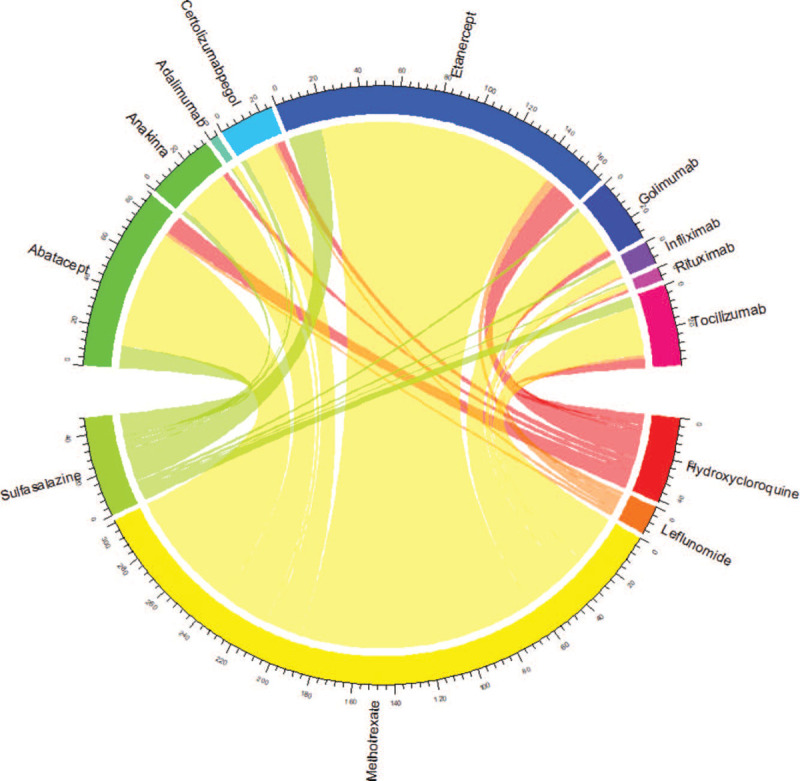
Chord diagram describing switch from cs-DMARD to b-DMARD in the first year. b-DMARDs = biological-DMARDs, cs-DMARDs = conventional synthetic-DMARDs.

In our cohort, in the first year of treatment, we observed a percentage of switch from cs-DMARDs to b-DMARDs of 7.9% with a median time for the first switch equal to 5 months. Moreover, new users of cs-DMARD changed on average 5 synthetic drugs before switching to b-DMARD. Regarding the switch from cs-DMARDs to b-DMARDs in the first year we observed that methotrexate was replaced by etarnecept, abatacept, and anakinra more frequently, with an important residual share of switch to other biologicals, anyhow (Fig. 3).

## 4 Discussion

In Italy, in clinical practice, >7% of patients with RA are prescribed with a b-DMARD as first-line treatment. This therapeutic option is not supported by guidelines, that recommend instead starting with cs-DMARDs, specifically with methotrexate, which is often described as the “anchor” drug for RA.^[17]^

However, the threshold for initiating more costly biologic DMARD therapy is not as clear cut and should be influenced by several factors, such as sociodemographic, physician, and insurance coverage factors.^[18,19]^

Our findings also show that younger age is the main determinant for first-line biological treatment followed by cancer, cardio-cerebrovascular diseases, and previous use of NSAIDs or corticosteroids.

In particular, the decision to administer biologics in DMARD-naïve patients among younger patients could reflect a perceived difference in efficacy between conventional systemic and biological DMARDs.

From the literature it is generally accepted that therapy with DMARDs should be started as soon as the diagnosis of RA is made, and that early control of inflammation results in a better outcome in terms of joint damage, functional status, and quality of life. Currently, patients with RA usually start with cs-DMARDs and switch to biological treatment if the therapeutic target is not met by optimal conventional treatment. Even if a novel treatment paradigm with biologic use in the early phase of RA has been proposed,^[20]^ the possibility that biological drugs might better control the disease in this phase of RA is unclear and supported by scarce evidence.^[21]^ Furthermore, although several biologics have demonstrated good efficacy and tolerability in short-term trials, a lack of robust long-term safety data requires more attention to this issue.

Evidently, the harms of biologics must be balanced against their benefits, when making a risk–benefit assessment of its use for a patient with systemic autoimmune conditions such as RA.^[22]^ Additionally, biological agents are expensive and compel physicians to consider the economic burden of the treatment choice. In fact, the use of biological drugs out of guide line indications needs to take in consideration both the risk of adverse events in long-term use and Italian National Health System sustainability. The association between cardio-cerebrovascular diseases and initiation of biologic DMARDs may reflect a good safety practice, in fact based on a recent meta-analysis an increased risk of cardiovascular events and stroke was observed in patients treated with cs-DMARDs compared with tumor necrosis factor inhibitors.^[23]^ To our knowledge, determinants in first-line use of b-DMARD in DMARDs naïve patients with RA have not previously investigated, however previous studies, in different countries, have shown that increasing age is associated with reduced chances of receiving biologics compared with younger patients, despite higher disease activity levels.^[18,24–26]^. Our findings show that the use of b-DMARDs as first-line include anti-TNF therapies, mostly as monotherapy, although a recent meta-analysis has clearly demonstrated that combining methotrexate with biological drug therapy in early RA achieved significantly better results.^[27]^

Additionally, in the first year of treatment we observed a percentage of switch from cs-DMARDs to b-DMARD equal to 7.9%. The median time of switch from the first DMARD prescription was 5 months. This window is in line with the EULAR guide line that suggests to monitor patients frequently during the active disease phase and, if there is no improvement by at most 3 months after the start of treatment or the target has not been reached by 6 months, therapy should be adjusted.

The observational nature of our study implies several limitations, in particular disease severity and patient clinical parameters are not recorded in administrative claim. A patient's disease activity could influence physician's decision in favor of a biologic agent as first-line therapy, even if guidelines suggest to start with a conventional systemic drug independently from disease severity. Moreover, the misclassification of first-line treatment due to a lack of data on out-of-pocket drug purchase and out of region prescriptions is possible. However, another study in the context of RA showed a similar use of b-DMARDs as first-line.^[28]^ Finally, our analysis is based on data from one single Italian central region and may not reflect clinical practice in other geographical areas.

## 5 Conclusion

Our findings show a use of b-DMARDs in RA patients not aligned with guidelines, both in terms of first-line treatment and monotherapy. Further analyses on real world data are necessary to investigate these issues and to promote a better use of biological drugs. The knowledge of RA management in a real-life clinical setting tracking separately DMARDs prescription by hospitals and local heath authority could offer an opportunity to improve the management of RA in Italy.

## Author contributions

**Conceptualization:** Antonio Addis, Nera Agabiti, Marina Davoli, Valeria Belleudi.

**Data curation:** Laura Angelici.

**Formal analysis:** Laura Angelici.

**Methodology:** Laura Angelici, Valeria Belleudi.

**Supervision:** Antonio Addis, Nera Agabiti, Marina Davoli, Valeria Belleudi.

**Writing – original draft:** Laura Angelici, Antonio Addis, Nera Agabiti, Ursula Kirchmayer, Valeria Belleudi.

**Writing – review & editing:** Laura Angelici, Nera Agabiti, Valeria Belleudi.
